# Supplementary material for: A mating-induced reproductive gene promotes Anopheles tolerance to Plasmodium falciparum infection
Source: PLoS Pathog. 2020 Dec 21;16(12):e1008908. doi: 10.1371/journal.ppat.1008908 (PMC7785212; doi:10.1371/journal.ppat.1008908)
Supplement: S1 Table — Generalized Linear Mixed Models (GLMMs) were constructed and analyzed in R. The test/model used in each figure and for each comparison is outlined with individual likelihood ratio test (LRT) outputs. Significant tests (p < 0.05) are bolded. (PDF) [file ppat.1008908.s001.pdf]

S1 Table

Figure	Response variable	Model	Effect Test Outputs
**1A**	egg number	GLMM, Gaussian distribution	mating: LRT X^2_1 = 0.009, p = 0.93<br>infection: LRT X^2_1 = 0.76, p = 0.38<br>mating*infection: LRT X^2_1 = 3.1, p = 0.08
**1B**	egg prevalence	GLMM, binomial distribution	**mating: LRT X^2_1 = 16.26, p < 0.001**<br>infection: LRT X^2_1 = 3.24, p = 0.07<br>mating*infection: LRT X^2_1 = 0.0001, p = 0.99
	egg number	GLMM, Gaussian distribution	**mating: LRT X^2_1 = 20.22, p < 0.001**<br>infection: LRT X^2_1 = 0.007, p = 0.94<br>mating*infection: LRT X^2_1 = 0.99, p = 0.32
**2A**	oocyst prevalence	GLMM, binomial distribution	mating: LRT X^2_1 = 1.08, p = 0.30
	oocyst intensity	GLMM, zero-truncated negative binomial distribution	mating: LRT X^2_1 = 0, p = 1.00
**2B**	oocyst prevalence	GLMM, binomial distribution	mating: LRT X^2_1 = 0.007, p = 0.93
	oocyst intensity	GLMM, zero-truncated negative binomial distribution	mating: LRT X^2_1 = 1.47, p = 0.23
**3A**	egg number	GLMM, zero-inflated negative binomial distribution	treatment: LRT X^2_1 = 0.16, p = 0.69<br>gametocytemia: LRT X^2_3 = 6.33, p = 0.10<br>**treatment*gametocytemia: LRT X^2_3 = 16.15, p = 0.001**
**3B**	oocyst prevalence	GLMM, binomial distribution	treatment: LRT X^2_1 = 0.60, p = 0.44<br>gametocytemia: LRT X^2_3 = 5.78, p = 0.12<br>treatment*gametocytemia: LRT X^2_3 = 4.63, p = 0.20
	oocyst intensity	GLMM, zero-truncated negative binomial distribution	treatment: LRT X^2_1 = 0.85, p = 0.36<br>**gametocytemia: LRT X^2_3 = 20.36, p < 0.001**<br>treatment*gametocytemia: LRT X^2_3 = 0.85, p = 0.84
**3C**	egg number	GLMM, zero-inflated negative binomial distribution	treatment: LRT X^2_1 = 0.08, p = 0.78<br>oocyst number: LRT X^2_1 = 1.89, p = 0.17<br>**treatment*oocyst number: LRT X^2_1 = 7.77, p = 0.005**
**3D**	egg prevalence	GLMM, binomial distribution	treatment: LRT X^2_1 = 0.06, p = 0.81<br>oocyst number: LRT X^2_1 = 1.07, p = 0.30<br>**treatment*oocyst number: LRT X^2_1 = 7.18, p = 0.007**
