# Supplementary material for: A mating-induced reproductive gene promotes Anopheles tolerance to Plasmodium falciparum infection
Source: PLoS Pathog. 2020 Dec 21;16(12):e1008908. doi: 10.1371/journal.ppat.1008908 (PMC7785212; doi:10.1371/journal.ppat.1008908)
Supplement: S2 Table — Samples of P. falciparum-infected blood were collected from 5 gametocyte donors and fed to An. coluzzii mosquitoes silenced for MISO or a control gene. The number of gametocytes for each sample was counted per 1000 white blood cells on blood smears, then converted to the number of gametocytes per μl of blood assuming a standard white blood cell count of 8000 cells per μl of blood. The outcome of infection was determined by counting the number of oocysts developed per female 7 d pIBF. (PDF) [file ppat.1008908.s002.pdf]

S2 Table

Donor	Gametocytes (/µl of blood)	dsRNA	Sample size	Oocyst intensity range	Oocyst intensity mean	Oocyst intensity median	Prevalence of infection (%)
1	104	ds*Control*	13	0-15	6.1	5.0	69.2
		ds*MISO*	31	0-22	5.5	3.0	71.0
2	304	ds*Control*	34	0-178	30.9	16.0	76.5
		ds*MISO*	24	0-127	33.5	17.5	75.0
3	72	ds*Control*	19	0-22	4.2	2.0	73.7
		ds*MISO*	20	0-30	8.5	5.0	95.0
4	128	ds*Control*	22	0-87	26.3	27.0	90.9
		ds*MISO*	18	0-62	27.2	24.5	83.3
5	72	ds*Control*	36	0-17	5.2	3.5	83.3
		ds*MISO*	7	1-11	4.6	3.0	100.0
