# Supplementary material for: A mating-induced reproductive gene promotes Anopheles tolerance to Plasmodium falciparum infection
Source: PLoS Pathog. 2020 Dec 21;16(12):e1008908. doi: 10.1371/journal.ppat.1008908 (PMC7785212; doi:10.1371/journal.ppat.1008908)
Supplement: Attachment — Submitted filename: Letter to Reviewers_Marcenac et al.docx [file ppat.1008908.s003.docx]

Boston, October 28, 2020

Dear Editor,

We are pleased to submit our revised manuscript entitled “**A mating-induced reproductive gene promotes *Anopheles* tolerance to *Plasmodium falciparum* infection**” for consideration for publication in *PLoS Pathogens*. We are grateful for the careful review that our original manuscript received. We have addressed all reviewers’ comments below, and we hope that with the revisions we have made to our manuscript, our work will be acceptable for publication in *PLoS Pathogens*.

**PART I - SUMMARY**

**Reviewer #1: This study examined whether P. falciparum interferes with egg development in mated An. gambiae and An. stephensi females. The study builds up on earlier work from the same group (Werling et al. 2019) that found a positive correlation between egg numbers and parasite loads in virgin females. Although this result suggested a non-competitive interaction between P. falciparum and An. gambiae, it remained to be determined whether this finding extended to reproductively active, mated females and other Anopheles species. The fitness cost of malaria parasite infection in mosquitoes is a relevant and longstanding question in vector biology.
Overall, I found this manuscript to be compelling and well written, and I think it will be of great interest to both vector biologists and a more general audience of evolutionary biologists. I have a few comments about the interpretation and discussion of the results, but with these relatively minor considerations taken into account, I would not hesitate to recommend this manuscript for publication.**

We thank Dr. Lambrechts (Reviewer #1) for his comments. We have addressed his comments on the interpretation and discussion of the results below, and we have made modifications to the text to reflect his feedback. We are glad and grateful that he found the work compelling and of great interest to vector biologists and evolutionary biologists.

**Reviewer #2: In this manuscript Marcenac et al. provide new information on reproduction and tolerance to Plasmodium infection. Through the use of reproduction/mating bioassays coupled with functional and infection experiments the authors provide convincing evidence that the MISO gene is involved in mosquito tolerance to Plasmodium infection, allowing it to maintain reproductive fitness.
The manuscript adds much needed new knowledge to understand the effects of Plasmodium infection on mosquito reproductive fitness and provides some potential mechanistic evidence for the absence of detrimental effects of Pf infection in the most important Anopheline vectors. The manuscript is easy to read and well organized when presenting the data via graphs or during the discussion section. I commend the authors for providing alternative hypothesis, not shying away from writing other potential explanations of the observable data.**

We thank Reviewer #2 for their comments and their praise of the work, including our discussion of alternative hypotheses for our results. We are glad they found that the manuscript adds important knowledge to the field.

**PART II – MAJOR ISSUES: KEY EXPERIMENTS REQUIRED FOR ACCEPTANCE**

**Reviewer #1: (No Response)**

**Reviewer #2: I don't see any major experiment that needs to be conducted. The experiments appear solid.**

**PART III – MINOR ISSUES: EDITORIAL AND DATA PRESENTATION MODIFICATIONS**

**Reviewer #1: 1) My most significant criticism is about the primary phenotype examined to measure the fitness cost of P. falciparum infection. The number of developed eggs within the ovaries after a single blood meal is only a partial measure of the mosquito’s reproductive output. Ideally, not only the total number of eggs laid across multiple gonotrophic cycles throughout a female’s lifetime (fecundity) should have been considered, but also the viability of these eggs (fertility) should have been assessed to more accurately estimate the overall fitness. I suggest the authors tone down their claims about fitness and the fitness cost of infection, and better contextualize their findings. For example, the statement that “P. falciparum infections do not affect the reproductive fitness” (line 197) should explicitly mention the caveat that the phenotype examined (number of developed eggs within the ovaries) did not fully encapsulate fitness.**

**Following up on the above point, why were the females not allowed to lay their eggs in the experiments? Oocyst numbers could have been determined after oviposition to obtain paired fecundity and parasite load measurements. Interfering with the natural process and time frame of oviposition may have altered life-history and resource allocation trade-offs. The authors may also consider justifying/discussing this point.**

We agree that the lack of fertility data is a limitation in our work. Unfortunately, due to logistical constraints during our field studies in Burkina Faso, we were unable to collect fertility data from individual females. In these conditions, when females are placed into oviposition cups, the rate of oviposition can be low while the rate of mortality is high, and so including fertility data would have substantially reduced our sample size. While preventing females from ovipositing could have interfered with natural processes, we observed that none of the females showed evidence of egg resorption when they were dissected at the time of oocyst counting. We have toned down our statements about the fitness costs of infection by qualifying that our assays measured only fecundity after one blood feeding as the fitness output (lines 21, 103-104, 114, 119, 195, 199, and 225). We have also added a section in the discussion highlighting that future work will need to assess other components of mosquito fitness, including fertility, egg development over multiple gonotrophic cycles, and survival (lines 216-219).

**2) Another aspect that may deserve to be discussed further relates to the concept of host tolerance. Tolerance is often represented as the negative slope of host fitness as a function of parasite burden. Individuals with a shallower slope are more tolerant because they maintain higher fitness at high parasite loads. In the present study, the relationship between the probability of egg development and the number of oocysts was negative in MISO-depleted individuals, consistent with a relatively lack of tolerance, however the same relationship had a positive slope in the control mosquitoes (Fig. 3D). Could this positive slope reflect the cost of resistance of individuals with low parasite numbers, if females who are able to clear the infection are less likely to produce eggs ? Or to the contrary, a positive effect of high parasite loads on the probability of egg development?**

We thank Dr. Lambrechts for these insights and agree that this point should be discussed further in our manuscript. Our current thinking given our results here and in Werling, Shaw, Itoe *et al.* 2019 is that it is not the *P. falciparum* parasite that dictates female fecundity, but rather, that processes that favor egg development also favor parasite survival. With regards to the fact that in Figure 3D, we saw that females that had a higher likelihood of egg development also harbored high oocyst loads, we hypothesize this could be because 1) females prioritized resources toward egg development, leaving few resources for an immune response against the parasite; or because 2) the processes regulated by blood feeding that induce egg development also benefit parasite survival, while not being specifically tailored to the parasite. From this feedback from Dr. Lambrechts, we realize that our sentence “Moreover, in *An. coluzzii* infections using natural *P. falciparum* isolates, we found that infections with increasing intensity did not induce a decrease in egg numbers, but rather increased the likelihood of egg development among control mated females” (previously lines 199-201 in the Discussion) may be misleading by suggesting causality whereby the parasite induced this fecundity phenotype. We have rewritten the sentence to better reflect our thinking (lines 204-206), and we have added a section to the discussion (lines 209-216) to provide hypotheses for our data in control females with high parasite loads.

**In the same line, the previous work from this group (Werling et al. 2019) found a positive relationship between egg and parasite numbers in laboratory assays. Because this specific result was not confirmed in the field-based experiments of the present study (Fig. 3C), it would be interesting to discuss the potential reasons underlying this discrepancy.**

Both control and ds*MISO* *An. coluzzii* females used in our experiments in Burkina Faso had higher rates of a failure to develop eggs compared to the mosquito strains we usually use in our laboratory experiments, which is consistent with pre-gravid behavior observed in natural anopheline populations (Charlwood *et al. Malaria Journal* 2003; Lyimo and Takken *Medical and Veterinary Entomology* 1993). This skewed our egg number distributions in a way that we could not employ Spearman’s correlations to look at the relationship between eggs and oocysts as done in Werling, Shaw, Itoe *et al.* 2019, and so could not specifically confirm this trend in our field experiments. We believe, however, that our analyses in Figure 3D expand on the results of Werling, Shaw, Itoe *et al.* 2019, because the higher rate of zero eggs in controls and ds*MISO* allowed us to assess the probability of egg development across the range of oocyst numbers in each treatment group.

**3) The statistical analyses were performed according to very high standards, however it would have been useful to specify how the error distribution was chosen in the various models.**

We apologize for this omission. For each of the models, we chose the most appropriate distribution through Akaike information criteria (AIC) comparisons. We have added this information on AIC comparisons to our Materials and Methods section (lines 432-433).

**Also, why were oocyst prevalence and intensity analyzed by separate GLMMs, whereas zero and non-zero egg counts were analyzed together?**

Our rationale for analyzing oocyst prevalence and intensity separately is that infection prevalence is a key metric in *P. falciparum* transmission and is measured directly within the Ross-MacDonald model, while infection intensity is measured indirectly via the likelihood of transmission. The parasite’s fitness, and thus transmissibility, is more deeply impacted by whether it can establish even a single oocyst in a female mosquito. Analyzing oocyst prevalence and intensity separately also allowed us to find that *P. falciparum* parasites were able to establish infection within the mosquito even at low gametocytemia, suggesting that gametocytemia and infection prevalence are not necessarily linked (at least within the gametocytemia range of our study). We have added a sentence in our results (lines 180-181) to highlight this result. Analyzing infection prevalence and intensity separately also allows us to remain consistent with recent works that we reference in our manuscript (Werling, Shaw, Itoe *et al.* 2019, Lawniczak *et al.* 2019).

Our rationale for the egg data analysis was that in the field experiments, separating out females that failed to develop eggs from females that did develop eggs would mean excluding from our analyses those females with the most profound fitness effect observed across the range of *P. falciparum* infections. We reasoned that in graphing out eggs and oocysts to detect a possible tolerance effect we should include females that fail to develop eggs. As a result, in our models we chose to analyze females with zero and non-zero eggs together, using GLMMs with zero-inflated negative binomial distributions.

**Reviewer #2: It would be good if the authors describe how many independent experiments were conducted and represented in each graph.**

The data from each figure is from 3 independent replicates, and we have now added the replicate size in the figure legends of all three figures to address this comment.

**Also, I am curious to know how the authors decided to employ Tukey HSD to do pairwise analysis. One of the assumptions for using Tukey HSD is that of equal sample sizes for each treatment; but on the graphs (in particular 2B) has vastly different sample sizes. Perhaps I am misunderstanding something here?**

We thank Reviewer #2 for their comment, as we now realize we need to better describe the analysis we did for pairwise comparisons. We used the function "emmeans" from the “emmeans” package (Reference 55). This function computes estimated marginal means and automatically corrects for unequal sample sizes using the Tukey-Kramer method. We have changed our figure legends to better reflect our statistical methods, and have added this information to our “Quantification and statistical analysis” section in the Materials and Methods (lines 454-456).

We thank you for considering our manuscript for publication in *PLOS Pathogens*.

Dr. Flaminia Catteruccia

Professor of Immunology and Infectious Disease

Harvard T. H. Chan School of Public Health

fcatter@hsph.harvard.edu

tel: +1 617 432 1773
